# Supplementary material for: MetaRibo-Seq measures translation in microbiomes
Source: Nat Commun. 2020 Jun 29;11:3268. doi: 10.1038/s41467-020-17081-z (PMC7324362; doi:10.1038/s41467-020-17081-z)
Supplement: Supplementary file 10 — Supplementary Data 7 [file 41467_2020_17081_MOESM10_ESM.zip › File2/Confidence_VeryHigh_Taxonomy/123165_out.krona.html]

Javascript must be enabled to view this page.

members
magnitude
magnitudeUnassigned
count
unassigned
taxon
rank

123165\_out

7

2
7
superkingdom

976
phylum
7

200643
class
7

order
7
171549

7
family
171552

7
genus
838

159272
species

SRS017849\_contig\_number\_contig-100\_4482.4482SRS049896\_contig\_number\_12088SRS049959\_contig\_number\_contig-100\_4570.204227
3

species

SRS1041031\_contig\_number\_30333
1
1263102

species

SRS012849\_contig\_number\_24499SRS1041116\_contig\_number\_6964SRS1041140\_contig\_number\_13698
3
165179
